# Regulatory Effects of Different Doses of Penoxsulam on Endogenous Hormones and Antioxidant System in Foxtail Millet

**DOI:** 10.3390/plants14213254

**Published:** 2025-10-24

**Authors:** Chunyan Hu, Tingting Chen, Chunxia Diao, Binglan Dou, Suqi Shang, Shuo Li, Yinyuan Wen, Xi’e Song, Juan Zhao, Hui Cao, Shuqi Dong

**Affiliations:** 1 College of Plant Protection, Shanxi Agricultural University, Jinzhong 030800, China; hucy4216@sxau.edu.cn (C.H.); fhldcx@163.com (C.D.); doubl0902@163.com (B.D.); 2 College of Agriculture, Shanxi Agricultural University, Jinzhong 030800, China; sxndctt@163.com (T.C.); ssq2025@163.com (S.S.); leesure2025@163.com (S.L.); wenyinyuan@126.com (Y.W.); sxndsxe@163.com (X.S.); sxndzhaojuan@163.com (J.Z.); 3 Special Orphan Crops Research Center of the Loess Plateau, MARA, Shanxi Agricultural University, Jinzhong 030800, China

**Keywords:** penoxsulam, foxtail millet, endogenous hormones, antioxidant system, correlation analysis

## Abstract

The specific objectives include three points: (1) to clarify the dynamic change laws of the contents of three key endogenous hormones, namely, indole-3-acetic acid (IAA), gibberellin (GA), and abscisic acid (ABA), in foxtail millet leaves after penoxsulam treatment, and their correlations with drug dose and treatment time; (2) to analyze the effects of different doses of penoxsulam on the antioxidant system of foxtail millet, specifically including the change characteristics of hydrogen peroxide (H_2_O_2_), superoxide anion (O_2_^−^), reduced glutathione (GSH) content, and cell membrane permeability (MP); and (3) to reveal the correlation between endogenous hormone changes and antioxidant system indicators through correlation analysis so as to provide a direct experimental basis for the screening of safe doses of penoxsulam application in foxtail millet fields and the research on the herbicide stress resistance mechanism of foxtail millet. Using Jingu 21 as the test material, four penoxsulam dose levels were set through pot and field experiments. The changes in endogenous hormone content, antioxidant system indexes, and phenotypic indicators of foxtail millet were determined at different periods after treatment, and the correlation between endogenous hormones and antioxidant systems was analyzed. Compared with the control (P_0_), the contents of IAA and GA in foxtail millet showed a “first increasing and then decreasing” trend, while the content of ABA showed a continuous increasing trend. With the increase in penoxsulam concentration, the contents of H_2_O_2_, O_2_^−^, GSH, and MP in foxtail millet gradually increased. A correlation analysis showed that there was a significant correlation between leaf endogenous hormones and the defense capacity of the antioxidant system. After penoxsulam treatment, foxtail millet leaves showed dynamic changes of “first increasing and then decreasing” in IAA and GA contents, and a continuous increase in ABA contents. At the same time, H_2_O_2_, O_2_^−^, GSH content, and MP increased significantly with the increase in the drug dose. It is speculated that foxtail millet may indirectly regulate the defense ability of the antioxidant system by regulating the content of endogenous hormones to alleviate the damage of herbicide stress.

## 1. Introduction

In recent years, the extensive application of pesticides has brought severe environmental problems, among which sulfonamide herbicides are widely used in various crop fields due to their remarkable weeding effect [1]. However, many studies have shown that the residues of sulfonamide substances in soil can cause harm to the environment, posing potential risks to ecological balance and subsequent crop growth [2,3,4].

Penoxsulam, a triazolopyrimidine herbicide, exerts its herbicidal effect by inhibiting acetolactate synthase (ALS) [5,6]. As a herbicide with a broad weed control spectrum in paddy fields, it can effectively control *Echinochloa* spp., annual *Cyperaceae* weeds, and most broad-leaved weeds in paddy fields. Moreover, it has the advantages of low application rate and long duration (30–60 days) [7], making it widely used in paddy field weed control. However, there are few reports on its application in foxtail millet fields, which limits the scientific formulation of weed control strategies in foxtail millet production. At present, weed control in foxtail millet fields still relies highly on a limited number of herbicides, such as prometryn, sethoxydim, bromoxynil octanoate, and monosulfuron [8]. Long-term and sole use of these herbicides has led to an increasingly prominent problem of weed resistance, so there is an urgent need to develop new, high-efficiency, and safe herbicide varieties to enrich control methods. Although penoxsulam occupies a dominant position in weed control in paddy fields, its application effect, safety, and physiological impact in foxtail millet fields remain unclear, and it is far from being a dominant herbicide in production practice. Therefore, evaluating the physiological stress effect of penoxsulam on foxtail millet and clarifying its safe application dose have important theoretical value and practical significance for expanding its application scope and scientifically assessing its potential in the weed management system of foxtail millet fields.

Endogenous hormones are key substances regulating crop growth and development, and play a core role when crops are subjected to adverse conditions. They can not only regulate plant growth and development processes, but also adjust the interaction between plants and the environment, induce the expression of crop defense genes, and enhance stress resistance [9,10]. When plants are under herbicide stress, the homeostasis of endogenous hormones in the body will be significantly disrupted. Among them, IAA is the main natural auxin in higher plants and regulates various growth and development processes [11]; GA can promote seed germination, break dormancy, induce flower bud formation and flowering, and enhance plant resistance to adversity [12]; and ABA is the core hormone for plants to respond to abiotic stress and can cope with various abiotic stresses by regulating growth and development [13].

Reactive oxygen species (ROS), including superoxide anion (O_2_^−^), H_2_O_2_, hydroxyl radical, and singlet oxygen, are products of plant physiological metabolism [14,15,16]. Under normal non-stress conditions, the production of ROS and the elimination of ROS by the antioxidant system are in a balanced state, and ROS will not accumulate. However, when plants are subjected to adversity stress such as herbicide application, ROS are overproduced, and the antioxidant system cannot eliminate ROS in time, leading to the disruption of this balance and the accumulation of ROS [17,18]. The excessive accumulation of ROS can damage the cell membrane system, affect the normal material exchange and energy transfer of plants, and even inhibit plant growth and development. The antioxidant system of plants mainly includes enzymatic and non-enzymatic antioxidant components. GSH, as a key component of the non-enzymatic antioxidant system in the ascorbate–glutathione (AsA-GSH) cycle, has the functions of scavenging ROS and maintaining intracellular homeostasis, and its content is closely related to plant stress resistance [19,20,21]. In addition, cell membrane permeability (MP) is an important indicator reflecting the integrity of the cell membrane system. When the cell membrane is damaged by stress, MP will increase, resulting in the extravasation of intracellular substances [22].

Foxtail millet is an important gramineous food crop in China, with the characteristics of drought resistance, salt tolerance, and low fertilizer requirement. Due to its small genome, short growth period, high seed yield, and ease of laboratory research, it has become a C4 model crop [23]. Studying the response mechanism of foxtail millet to abiotic stress is of great significance for improving its stress resistance and ensuring stable yield. Constructing a scientific and efficient weed control system in grain fields has become a key technical demand to ensure food security and enhance industrial benefits [24]. At present, there is a lack of in-depth research on the effects of penoxsulam on the endogenous hormone metabolism and antioxidant system of foxtail millet, and the relationship between endogenous hormones and the antioxidant system under penoxsulam stress remains unclear.

Therefore, this study takes the widely planted conventional high-quality foxtail millet variety “Jingu 21” as the test material, and combines pot and field experiments to set four different penoxsulam treatment doses (0, 15, 30, and 60 g a.i. Ha^−1^). The objectives of this study are to (1) clarify the dynamic change laws of the contents of three key endogenous hormones (IAA, GA, ABA) in foxtail millet leaves after penoxsulam treatment, and their correlations with drug dose and treatment time; (2) analyze the effects of different doses of penoxsulam on the antioxidant system of foxtail millet, including the change characteristics of H_2_O_2_, O_2_^−^, GSH contents and MP; and (3) reveal the correlations between endogenous hormone changes and antioxidant system indicators through correlation analysis so as to provide a direct experimental basis for the screening of safe application doses of penoxsulam in foxtail millet fields and the research on the herbicide stress resistance mechanism of foxtail millet.

## 2. Results

### 2.1. Effects of Penoxsulam Spraying on Endogenous Hormones of Foxtail Millet

Data from pot and field experiments showed that with the increase in the penoxsulam spraying dose, the IAA content of Jingu 21 showed a dynamic change trend of “first increasing and then decreasing”.

**Figure 1:**
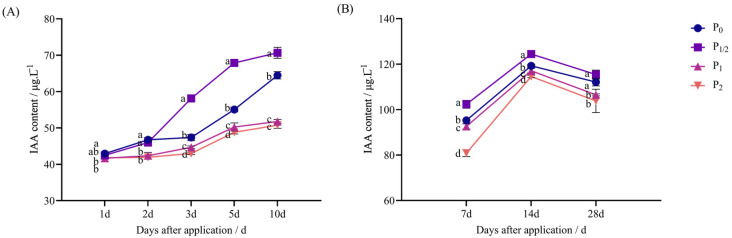
Effect of penoxsulam on IAA content of foxtail millet. (**A**) Pot experiment, (**B**) field experiment. Comparison between treatments of different concentrations on the same day, with lowercase letters representing a significant difference (*p* < 0.05). P_0_–P_2_ represent four different spraying doses of 0, 15, 30, and 60 g a.i. Ha^−1^, respectively.

Figure 1A shows the pot experiment: 1 d after herbicide application, the IAA content in the P_1/2_ treatment exhibited no significant difference from that of P_0_, while the P_1_ and P_2_ treatments decreased by 2.89% and 2.67% compared with P_0_. Then, 2 d after application, the P_1/2_ treatment exhibited no significant difference from P_0_, while the P_1_ and P_2_ treatments decreased by 9.39% and 10.33% compared with P_0_. Three days after application, the P_1/2_ treatment increased by 22.69% compared with P_0_, while the P_1_ and P_2_ treatments decreased by 5.64% and 9.23% compared with P_0_. Five days after application, the P_1/2_ treatment increased by 23.29% compared with P_0_, while the P_1_ and P_2_ treatments decreased by 8.73% and 11.37% compared with P_0_. Then, 10 d after application, the P_1/2_ treatment increased by 9.59% compared with P_0_, while the P_1_ and P_2_ treatments decreased by 19.71% and 21.25% compared with P_0_.

Figure 1B shows the field experiment: 7 d after application, the IAA content in the P_1/2_ treatment significantly increased by 7.39% compared with P_0_, while the P_1_ and P_2_ treatments decreased by 2.84% and 15.18% compared with P_0_, respectively. Fourteen days after application, the P_1/2_ treatment increased by 4.34% compared with P_0_, while the P_1_ and P_2_ treatments decreased by 1.81% and 3.92% compared with P_0_. Then, 28 d after application, the P_1/2_ treatment exhibited no significant difference from P_0_, while the P_1_ and P_2_ treatments decreased by 5.01% and 7.39% compared with P_0_.

After the application of penoxsulam in the pot and field experiments, the GA content of foxtail millet showed a “first increasing and then decreasing” trend with the increase in penoxsulam application dose after application.

**Figure 2:**
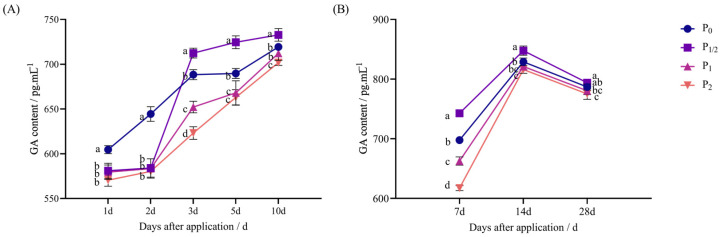
Effect of penoxsulam on GA content of foxtail millet. (**A**) Pot experiment, (**B**) field experiment. Comparison between treatments of different concentrations on the same day, with lowercase letters representing a significant difference (*p* < 0.05). P_0_–P_2_ represent four different spraying doses of 0, 15, 30, and 60 g a.i. Ha^−1^, respectively.

Figure 2A shows the pot experiment: 1 d after application, the GA content in the concentration treatments decreased by 3.91%, 4.17%, and 5.65% compared with P_0_. Two days after application, the concentration treatments decreased by 9.35%, 9.46%, and 9.94% compared with P_0_. Three days after application, the P_1/2_ treatment increased by 3.50% compared with P_0_, while the P_1_ and P_2_ treatments decreased by 5.25% and 9.47% compared with P_0_. Then, 5 d after application, the P_1/2_ treatment increased by 5.05% compared with P_0_, while the P_1_ and P_2_ treatments decreased by 3.14% and 3.88% compared with P_0_. Ten days after application, the P_1_ treatment exhibited no significant difference from P_0_. The P_1/2_ treatment increased by 1.86% compared with P_0_, and the P_2_ treatment decreased by 2.45% compared with P_0_.

As shown in Figure 2B, in the field experiment, 7 d after application, the P_1/2_ treatment increased by 6.46% compared with P_0_, while the P_1_ and P_2_ treatments decreased by 5.02% and 11.52% compared with P_0_. Fourteen days after application, the P_1/2_ treatment increased by 2.26% compared with P_0_. The P_1_ treatment exhibited no significant difference from P_0_, and the P_2_ treatment decreased by 1.56% compared with P_0_. Finally, 28 d after application, the P_1/2_ treatment exhibited no significant difference from P_0_, while the P_1_ and P_2_ treatments decreased by 0.79% and 1.48% compared with P_0_.

In both pot and field experiments, penoxsulam significantly increased the ABA content of foxtail millet, and it showed an increasing trend with the increase in the herbicide dose.

**Figure 3:**
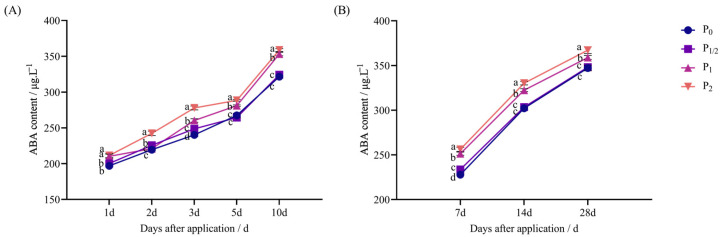
Effect of penoxsulam on ABA content of foxtail millet. (**A**) Pot experiment, (**B**) field experiment. Comparison between treatments of different concentrations on the same day, with lowercase letters representing a significant difference (*p* < 0.05). P_0_–P_2_ represent four different spraying doses of 0, 15, 30, and 60 g a.i. Ha^−1^, respectively.

As shown in Figure 3A, in the pot experiment, 1 d after application, the P_1/2_ treatment exhibited no significant difference from P_0_, while the P_1_ and P_2_ treatments increased by 6.78% and 7.33% compared with P_0_. Two days after application, the P_1_ treatment exhibited no significant difference from P_0_, while the P_1/2_ and P_2_ treatments increased by 2.84% and 10.41% compared with P_0_. Three days after application, the concentration treatments increased by 3.53%, 8.34%, and 15.64% compared with P_0_. Five days after application, the P_1/2_ treatment exhibited no significant difference from P_0_, while the P_1_ and P_2_ treatments increased by 5.10% and 7.91% compared with P_0_. Finally, 10 d after application, the P_1/2_ treatment exhibited no significant difference from P_0_, while the P_1_ and P_2_ treatments increased by 9.84% and 11.57% compared with P_0_.

Figure 3B illustrates the field experiment: 7 d after application, the concentration treatments increased by 2.57%, 10.32%, and 12.44% compared with P_0_. Then, 14 d after application, the P_1/2_ treatment exhibited no significant difference from P_0_, while the P_1_ and P_2_ treatments increased by 6.69% and 9.30% compared with P_0_. Finally, 28 d after application, the P_1/2_ treatment exhibited no significant difference from P_0_, while the P_1_ and P_2_ treatments increased by 3.41% and 5.77% compared with P_0_, respectively.

### 2.2. Effects of Penoxsulam Spraying on Antioxidant System of Foxtail Millet

After penoxsulam treatment, the H_2_O_2_ content of Jingu 21 in both pot and field experiments increased with the increase in the herbicide dose.

**Figure 4:**
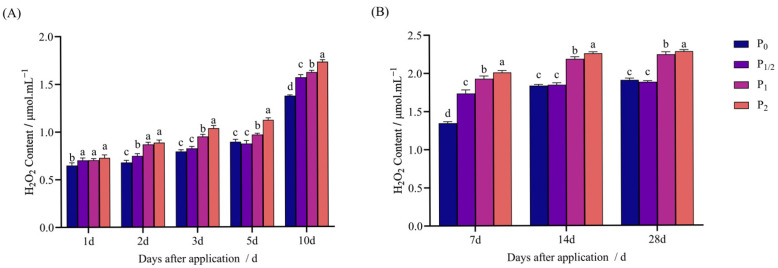
Effect of penoxsulam on H_2_O_2_ content of foxtail millet. (**A**) Pot experiment, (**B**) field experiment. Comparison between treatments of different concentrations on the same day, with lowercase letters representing a significant difference (*p* < 0.05). P_0_–P_2_ represent four different spraying doses of 0, 15, 30, and 60 g a.i. Ha^−1^, respectively.

As shown in Figure 4A, in the pot experiment, 1 d after application, the H_2_O_2_ content in the concentration treatments increased by 8.20%, 8.51%, and 12.20% compared with P_0_. Two days after application, the concentration treatments increased by 10.30%, 28.06%, and 30.71% compared with P_0_. Then, 3–5 d after application, the P_1/2_ treatment exhibited no significant difference from P_0_. Among them, 3 d after application, the P_1_ and P_2_ treatments increased by 19.83% and 30.75% compared with P_0_. Five days after application, the P_1_ and P_2_ treatments increased by 8.20% and 25.41% compared with P_0_. Ten days after application, the concentration treatments increased by 14.06%, 17.98%, and 25.88% compared with P_0_.

Figure 4B shows the field experiment: 7 d after application, the concentration treatments increased by 28.95%, 43.13%, and 49.25% compared with P_0_. Then, 14 d after application, the P_1/2_ treatment exhibited no significant difference from P_0_, while the P_1_ and P_2_ treatments increased by 19.12% and 23.08% compared with P_0_. Finally, 28 d after application, the P_1/2_ treatment had no significant difference from P_0_, while the P_1_ and P_2_ treatments increased by 17.56% and 19.59% compared with P_0_, respectively.

The O_2_^−^ content of the foxtail millet increased with the increase in the penoxsulam dose and decreased with the extension of the treatment time, but there was still a significant difference from P_0_.

**Figure 5:**
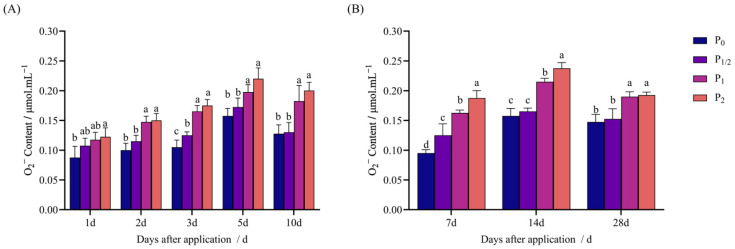
Effect of penoxsulam on O_2_^−^ content of foxtail millet. (**A**) Pot experiment, (**B**) field experiment. Comparison between treatments of different concentrations on the same day, with lowercase letters representing a significant difference (*p* < 0.05). P_0_–P_2_ represent four different spraying doses of 0, 15, 30, and 60 g a.i. Ha^−1^, respectively.

As shown in Figure 5A, in the pot experiment, 1 d after application, the P_2_ treatment increased by 21.14% compared with P_0_, while the P_1/2_ and P_1_ treatments exhibited no significant difference from P_0_. Two days after application, the P_1/2_ treatment exhibited no significant difference from P_0_, while the P_1_ and P_2_ treatments increased by 40.11% and 42.31% compared with P_0_. Three days after application, the concentration treatments increased by 36.31%, 84.08%, and 91.72% compared with P_0_. Five days application, the P_1/2_ treatment exhibited no significant difference from P_0_, while the P_1_ and P_2_ treatments increased by 41.82% and 55.45% compared with P_0_. Finally, 10 d after application, the P_1/2_ treatment exhibited no significant difference from P_0_, while the P_1_ and P_2_ treatments increased by 26.20% and 39.48% compared with P_0_.

Figure 5B shows the field experiment: 7 d after application, the concentration treatments increased by 26.63%, 68.05%, and 88.76% compared with P_0_. Fourteen days after application, the P_1/2_ treatment exhibited no significant difference from P_0_, while the P_1_ and P_2_ treatments increased by 36.76% and 51.10% compared with P_0_. Finally, 28 d after application, the P_1/2_ treatment exhibited no significant difference from P_0_, while the P_1_ and P_2_ treatments increased by 28.06% and 31.62% compared with P_0_.

In both pot and field experiments, the GSH content of Jingu 21 increased with the increase in the application rate after penoxsulam treatment and decreased with the extension of treatment time, but there was still a significant difference from P_0_.

**Figure 6:**
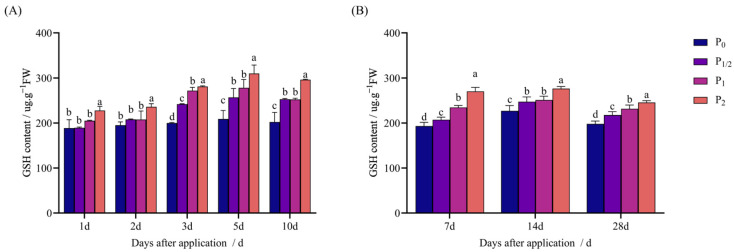
Effect of penoxsulam on GSH content of foxtail millet. (**A**) Pot experiment, (**B**) field experiment. Comparison between treatments of different concentrations on the same day, with lowercase letters representing a significant difference (*p* < 0.05). P_0_–P_2_ represent four different spraying doses of 0, 15, 30, and 60 g a.i. Ha^−1^, respectively.

Figure 6A shows the pot experiment: 1–2 d after application, the P_1/2_ and P_1_ treatments exhibited no significant difference from P_0_. The P_2_ treatment increased by 20.62% compared with P_0_ 1 d after application. The P_2_ treatment increased by 20.85% compared with P_0_ 2 d after application. Three days after application, the concentration treatments increased by 21.00%, 36.01%, and 40.61% compared with P_0_. Five days after application, the concentration treatments increased by 22.88%, 33.07%, and 48.24% compared with P_0_. Ten days after application, the concentration treatments increased by 25.03%, 24.78%, and 46.45% compared with P_0_, respectively.

As shown in Figure 6B, in the field experiment, 7 d after application, the P_1/2_ and P_1_ treatments increased by 7.35% and 21.28% compared with P_0_, respectively. The P_2_ treatment increased by 39.92% compared with P_0_. Fourteen days after application, the concentration treatments increased by 9.00%, 10.72%, and 21.96% compared with P_0_. Then, 28 d after application, the concentration treatments increased by 9.95%, 16.91% and 23.93% compared with P_0_, respectively.

In both pot and field experiments, the MP of foxtail millet increased with the increase in the spraying amount after penoxsulam treatment and the increase rate slowed down with time, but there was still a significant difference from P_0_.

**Figure 7:**
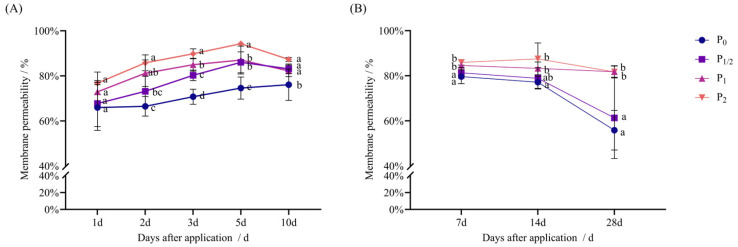
Effect of penoxsulam on membrane permeability of foxtail millet. (**A**) Pot experiment, (**B**) field experiment. Comparison between treatments of different concentrations on the same day, with lowercase letters representing a significant difference (*p* < 0.05). P_0_–P_2_ represent four different spraying doses of 0, 15, 30, and 60 g a.i. Ha^−1^, respectively.

Figure 7A shows the pot experiment: 1 d after application, the MP of each treatment exhibited no significant difference from P_0_. Two days after application, the P_1/2_ treatment exhibited no significant difference from P_0_, while the P_1_ and P_2_ treatments increased by 22.09% and 29.00% compared with P_0_. Three days after application, the concentration treatments increased by 13.43%, 20.08%, and 26.94% compared with P_0_. Five days after application, the concentration treatments increased by 15.29%, 16.64%, and 26.36% compared with P_0_. Ten days after application, the concentration treatments increased by 9.29%, 8.08%, and 14.72% compared with P_0_.

As shown in Figure 7B, in the field experiment, the MP of the P_1/2_ treatment exhibited no significant difference from P_0_. Seven days after application, the P_1_ and P_2_ treatments increased by 6.20% and 7.87% compared with P_0_. Fourteen days after application, the P_1/2_ and P_1_ treatments exhibited no significant difference from P_0_, while the P_2_ treatment increased by 13.44% compared with P_0_. Finally, 28 d after application, the P_1/2_ treatment exhibited no significant difference from P_0_, while the P_1_ and P_2_ treatments increased by 46.42% and 46.20% compared with P_0_.

### 2.3. Relationship Between Endogenous Hormone and Antioxidant System of Foxtail Millet

**Figure 8:**
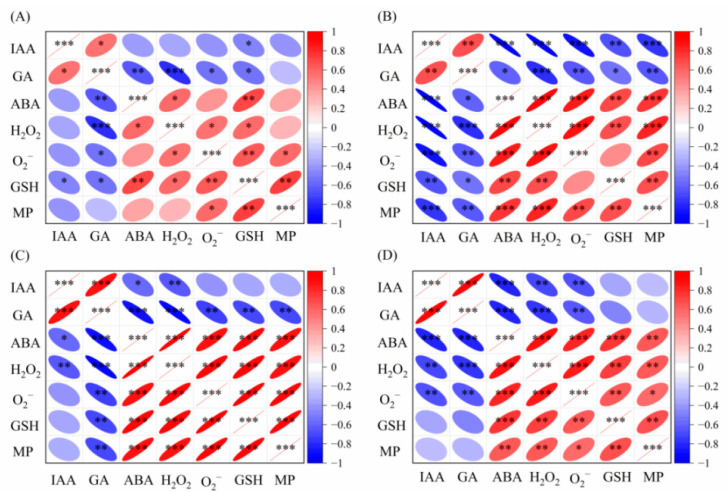

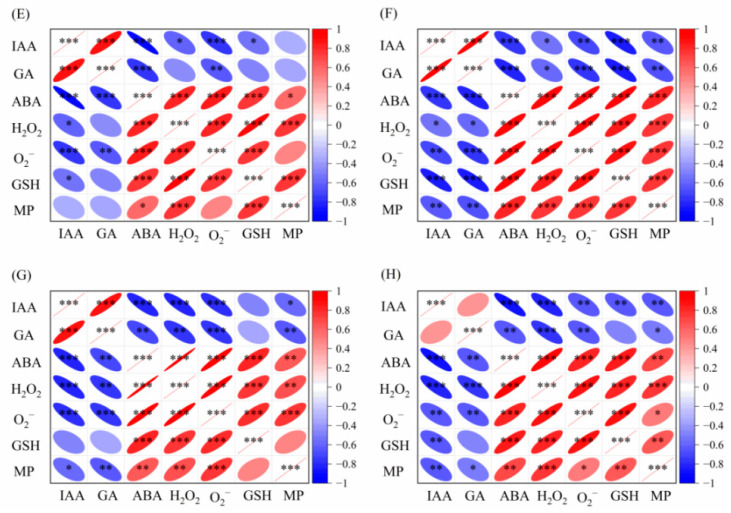
Correlation between endogenous hormones in the leaves and defense system of the antioxidant system. (**A**) Pot experiment, 1 d after treatment; (**B**) pot experiment, 2 d after treatment; (**C**) pot experiment, 3 d after treatment; (**D**) pot experiment, 5 d after treatment; (**E**) pot experiment, 10 d after treatment; (**F**) field experiment, 7 d after treatment; (**G**) field experiment, 14 d after treatment; (**H**) field experiment, 28 d after treatment. The color depth of the heatmap represents the correlation coefficient (red: positive correlation; blue: negative correlation); * *p* ≤ 0.05, ** *p* ≤ 0.01, *** *p* ≤ 0.001; each data point is the average of 4 biological replicates (pot experiment) or 4 field replicates (field experiment). All correlation analysis data are from the original determination values of this study.

The results of the correlation analysis (Figure 8) that 1 d after treatment, the IAA content was positively correlated with GA content, GA content was negatively correlated with antioxidant system-related indexes, and ABA content was significantly positively correlated with antioxidant system-related indexes (Figure 8A). Two days after treatment, the ABA content was positively correlated with antioxidant system-related indexes, while the IAA and GA contents were negatively correlated with antioxidant system-related indexes (Figure 8B). Three days after treatment, the GA content was negatively correlated with antioxidant system-related indexes, and ABA content was positively correlated with antioxidant system-related indexes (Figure 8C). Five days after treatment:, the IAA and GA contents were negatively correlated with H_2_O_2_ and O_2_^−^ contents, and the ABA content was positively correlated with antioxidant system-related indexes (Figure 8D). Ten days after treatment, the IAA content was negatively correlated with H_2_O_2_, O_2_^−^ and GSH contents, and the ABA content was positively correlated with antioxidant system-related indexes (Figure 8E).

Seven days after treatment (field), the IAA and GA contents were negatively correlated with antioxidant system-related indexes and the ABA content was positively correlated (Figure 8F). Fourteen days after treatment, the IAA and GA contents were negatively correlated with ABA content, and ABA content was positively correlated with antioxidant system-related indexes (Figure 8G). Finally, 28 d after treatment, the IAA content was negatively correlated and ABA content was positively correlated with antioxidant system-related indexes (Figure 8H).

## 3. Discussion

The plant hormone system can participate in the plant’s response to adversity by regulating growth, development, and environmental adaptability, which is usually the result of the synergistic effect of multiple hormones [9,25]. Previous studies have shown that low concentrations of ethametsulfuron reduce the ABA content and significantly increase the GA content in rice and soybean seedlings, while high concentrations significantly increase the ABA content and reduce the GA content [26]. When plants are subjected to adversity stress, they respond to stress by changing the content of endogenous hormones, and herbicide stress significantly affects the contents of IAA and GA in plants [27]. Mansour et al. [28] found that fluometuron and trifluralin treatments reduced the contents of IAA and ABA in maize and soybean tissues. Locher et al. [29] found that 5 μmol·L^−1^ trifluralin increased the ABA content in maize roots, but had no effect on the ABA content in mung bean roots. Li et al. [30] found that the ABA contents in the roots and stems of maize seedlings were directly proportional to the atrazine concentration.

The results of this study showed that, after penoxsulam treatment, the contents of IAA and GA in Jingu 21 showed a “first increasing and then decreasing” trend with the change in concentration, while the ABA content continued to increase. Low-concentration penoxsulam treatment can increase the contents of IAA and GA, showing a certain promoting effect on the growth of foxtail millet seedlings. High-concentration treatment leads to ABA accumulation and reduced GA content, weakening the antagonistic effect of GA on ABA accumulation and ultimately causing the abnormal yellowing of leaves. This hormone imbalance gradually recovered after 28 days of treatment, with the growth of the foxtail millet seedlings returning to normal.

Plants produce a large amount of ROS under stress conditions, and the excessive accumulation of ROS causes harm to plants [31], for example, destroying the cell membrane system, leading to an increase in relative conductivity. With the continuous accumulation of ROS, the increase in relative conductivity will further expand [32]. Cordon et al. [33] found that the accumulation of H_2_O_2_ in foxtail millet leaves under atrazine stress was 65.80% higher than that in the control. Amidosulfuron can induce the accumulation of H_2_O_2_ in non-target crop barley, causing oxidative damage [34]. Pan et al. [35] found that halosulfuron-methyl disrupts the H_2_O_2_ balance in soybeans, leading to serious oxidative damage. GSH is a tripeptide composed of glutamic acid, cysteine, and glycine, which can act as an oxidation signal in stressful environments to induce plants to increase the activity of antioxidant enzymes [36].

In this study, the content of H_2_O_2_ increased in a dose-dependent manner with the increase in the penoxsulam concentration, indicating that the foxtail millet suffered serious oxidative stress, which was consistent with the result found by Yuan et al. [37] that “the H_2_O_2_ level in Isatis indigotica increased with the increase of nicosulfuron treatment concentration”. In addition, penoxsulam treatment also significantly promoted the accumulation of O_2_^−^ in Jingu 21, leading to enhanced membrane lipid peroxidation, reduced plasma membrane stability, electrolyte leakage, and, ultimately, damage to foxtail millet [38,39]. At the same time, the GSH content increased with the increase in the penoxsulam dose after spraying; the increase rate slowed down with the extension of treatment time, but was still higher than that of the control. This indicates that, under long-term stress, foxtail millet is still in a state of adversity. Although the phenotype returned to normal, there was still ROS accumulation in the body, requiring the synergistic effect of enzymatic and non-enzymatic antioxidant factors to scavenge ROS.

Existing studies have confirmed that there is a two-way connection between the antioxidant enzyme system and hormone signaling pathways. The antioxidant enzyme system can affect plant development processes through concentration regulation, while hormones can regulate the scavenging and accumulation of antioxidant enzyme activities in plants [40]. This study found that the ABA content of endogenous hormones in foxtail millet leaves was positively correlated with H_2_O_2_, O_2_^−^, GSH content, and relative membrane permeability, while IAA and GA contents were negatively correlated with antioxidant system indexes, indicating that changes in IAA, GA, and ABA contents play a key role in plant resistance to adversity.

However, it should be noted that this study only established the correlation between endogenous hormones and antioxidant systems through correlation analysis, and the causal relationship between them needs to be further verified by follow-up experiments. From the perspective of “effectiveness”, as an ALS inhibitor, penoxsulam demonstrates excellent control efficiency against various gramineous and broad-leaved weeds, and its effectiveness has been validated in paddy fields. However, when applied to foxtail millet fields, “selectivity” becomes a key limiting factor. This study indicates that even penoxsulam at the recommended dosage can induce measurable oxidative stress and endogenous hormone imbalance in foxtail millet seedlings, with the stress effect being more pronounced at higher dosages. This suggests that the tolerance of foxtail millet to penoxsulam is limited. Therefore, although penoxsulam is “effective” in weed control, its safety to foxtail millet crops themselves determines that it cannot currently serve as a “dominant” herbicide in foxtail millet fields. Future research should focus on enhancing its selectivity by screening resistant varieties, developing safeners, or exploring precise application technologies, thereby unlocking its potential in supplementing the existing range of herbicide varieties for foxtail millet fields. For example, exogenous hormone supplementation experiments (spraying different concentrations of ABA) can be designed to determine the changes in antioxidant system indexes and phenotypic recovery rate of foxtail millet after spraying; or gene editing technology can be used to silence the key gene NCED in ABA synthesis, and determine the hormone content, antioxidant system indexes, and stress tolerance of silenced plants under penoxsulam treatment, so as to clarify the direct role of endogenous hormones in regulating the antioxidant system.

## 4. Materials and Methods

### 4.1. Materials

The tested herbicide was 25 g/L penoxsulam, provided by Dow Yinong Agricultural Science and Technology (Jiangsu) Co., Ltd., Nantong, China. The seeds of the tested foxtail millet variety “Jingu 21” were provided by the Institute of Economic Crops of Shanxi Agricultural University, Taiyuan, China.

### 4.2. Experimental Design

#### 4.2.1. Pot Experiment

Foxtail millet was cultivated using the greenhouse pot method. Thinning was conducted when the seedlings reached the 1–2-leaf stage, with 5–6 plants retained per pot. The seedlings were placed in an artificial climate chamber for cultivation, with the conditions set as a temperature of 25 °C, photoperiod of 16 h light/8 h dark, and watered with tap water during cultivation. When the foxtail millet grew to the 3–5-leaf stage, leaf samples from the control and treatment groups were collected for index determination.

Equipment: a 3WP-2000 Bioassay Spray Tower (developed by the Nanjing Institute of Agricultural Mechanization, Ministry of Agriculture and Rural Affairs, Nanjing, China) was used for foliar spraying, and the application rate is shown in Table 1. The control (P_0_) was sprayed with the same amount of clear water.

Sample collection time: 1 d, 2 d, 3 d, 5 d, and 10 d after herbicide application.

Location: Crop Chemical Regulation Laboratory, College of Agriculture, Shanxi Agricultural University.

#### 4.2.2. Field Experiment

The experiment was conducted from May to September 2024, and field management such as sowing, fertilization, and irrigation was carried out in accordance with local conventional standards. The nutrient content of the soil layer before sowing is shown in Table 2.

Equipment: A manual knapsack sprayer (working pressure of 300 kPa) with a fan-shaped nozzle was used. After calibration, the liquid application rate was 450 L/ha, and the application rate was the same as in Table 1.

Sample collection time: 7 d, 14 d, and 28 d after herbicide application.

Location: Agricultural Crop Station, College of Agriculture, Shanxi Agricultural University.

### 4.3. Determination Indexes and Methods

#### 4.3.1. Determination of Endogenous Hormone Contents

Referring to the method of Zhao et al. [41], 0.5 g of foxtail millet seedling samples were weighed, and the enzyme-linked immunosorbent assay (ELISA) was used for the quantitative analysis of endogenous hormone contents in different treatment groups.

Ⅰ. Standard curve preparation: Standard solutions were prepared with 5 concentration gradients according to the kit instructions, and 50 μL of each gradient was taken for duplicate well loading.

Ⅱ. Sample pretreatment: Plate hole setting: Blank control wells and sample detection wells were set up. Sample filling: 40 μL of sample diluent was pre-filled to the detection wells, and 10 μL of the test sample was accurately pipetted and injected vertically into the bottom of the well to avoid contact with the well wall. The sample was gently shaken and mixed evenly.

Ⅲ. Immunoreaction process: Primary incubation—after sealing, the samples were incubated at 37 °C for 30 min. Washing procedure: preparation of working lotion—the concentrated solution was diluted with distilled water at a 1:30 ratio. The liquid was discarded and the plate patted to remove residues. Then, 300 μL/ hole washing liquid was allowed to stand, was cleaned for 30 s, and the process repeated for 5 cycles. After the last cleaning, the plate was patted dry. Enzyme-labeled reaction: Except for blank wells, 50 μL of enzyme-labeled reagent was added to each well accurately. The samples were sealed again and incubated at 37 °C for 30 min.

Ⅳ. Signal detection: Color reaction—50 μL of chromogen A and 50 μL of chromogen B were added to each well in sequence. The samples were incubated at 37 °C in the dark for 15 min, and gently shaken to mix evenly during this period. Reaction termination: 50 μL of stop solution was added to each well.

Ⅴ. Data collection and analysis: Within 15 min of the reaction being terminated, the OD_450_ value of each hole was determined using an enzyme-labeled instrument.

#### 4.3.2. Determination of H_2_O_2_ Content

Referring to the method described by Du et al. [42], a kit produced by Beijing Solarbio (Science & Technology Co., Ltd., Beijing, China) (catalog number BC3595) was used to determine the H_2_O_2_ content according to the instructions.

#### 4.3.3. Determination of O_2_^−^ Content

The content of O_2_^−^ was determined by the hydroxylamine oxidation method [43]: 0.1 g of fresh leaf tissue was accurately weighed and placed in a pre-cooled mortar. Then, 50 mmol/L of pre-cooled phosphate-buffered solution was added, and the sample was fully ground into homogenate under ice bath conditions before being centrifuged at 12,000× *g* at 4 °C for 15 min. Then, 500 μL of supernatant was transferred to a new centrifuge tube. A total of 50 mmol/L phosphate-buffered solution and 10 mmol/L hydroxylamine hydrochloride solution were added in sequence, vortexed to mix, and incubated at 25 °C for 20 min. Chromogenic reagents were added: 17 mM p-aminobenzenesulfonic acid solution and 7 mM α-naphthylamine solution, and the sample was developed in a constant-temperature water bath at 30 °C for 30 min. Then, 50 mmol/L phosphate-buffered solution was taken as the blank control. The absorbance of the sample was measured at a wavelength of 530 nm, and the data of three repeated measurements were recorded.

#### 4.3.4. Determination of GSH Content

The GSH determination method was conducted as per Sedlak [44], where 0.1 g of foxtail millet seedling leaves were weighed and ground with 1 mL of precooled trichloroacetic acid (containing 5 mmol/L Na_2_-EDTA) in an ice bath, and then centrifuged at 12,000× *g* for 20 min. Then, 100 μL of supernatant was taken and added to 1 mL of 0.1 mM phosphate-buffered solution (pH = 7.7) and 0.5 mL of 5,5′-Dithiobis (2-nitrobenzoic acid) (DTNB) solution. Phosphate-buffered solution was used as the blank control instead of DTNB reagent; the sample was oscillated and mixed at 30 °C for 5 min, and the absorbance value was measured at OD_412_.

#### 4.3.5. Determination of MP

The MP was determined with reference to the method of Wei et al. [45]: fresh leaves were washed with deionized water, dried with filter paper, cut, and mixed evenly. After weighing 0.2 g and adding 20 mL deionized water to immerse the leaves, the solution was shaken once every 20 min, and after soaking for 60 min, the initial conductivity S1 was determined using a conductivity meter. After sealing, the sample was boiled in a boiling water bath for 10 min to kill the plant tissue, taken out and cooled, and then the conductivity S2 was determined. The conductivity S0 of deionized water at room temperature was determined and the MP value calculated.

### 4.4. Data Processing

A completely randomized experimental design was adopted in this study, and all pot experiments were set with four biological replicates. Data collation and analysis were completed using Microsoft Excel 2021 (Microsoft, Redmond, WA, USA) and IBM SPSS Statistics 27 software (SPSS Inc., Chicago, IL, USA), respectively. During the statistical inference process, one-way analysis of variance (ANOVA) was performed on the data that met the homogeneity of variance. Duncan’s multiple range test was used to compare the differences between groups, with a significance level of *p* < 0.05. Correlation was assessed using Pearson’s correlation analysis. The complete raw experimental data related to the content in this paper are provided in detail in the Supplementary Materials. Graphs were plotted using GraphPad Prism 10 (GraphPad Software, LLC, San Diego, CA, USA) and Origin 2024 (OriginLab, Northampton, MA, USA).

## 5. Conclusions

Penoxsulam treatment can induce significant changes in endogenous hormone metabolism in foxtail millet. The contents of IAA and GA show a dynamic trend of “first increasing and then decreasing”, while the ABA content accumulates significantly. These changes break the dynamic balance of endogenous hormones and inhibit the normal growth and development of foxtail millet. After 28 days of application, the antagonistic–synergistic relationship between hormones is gradually re-established, and the homeostasis of endogenous hormones is restored, promoting the gradual recovery of the normal growth of foxtail millet seedlings. With the increase in the penoxsulam concentration, the contents of H_2_O_2_, O_2_^−^, GSH, and MP in Jingu 21 gradually increase, thereby reducing the damage caused by herbicides to the cell membrane structure and improving the resistance of foxtail millet to penoxsulam. The correlation analysis shows that the ABA content of endogenous hormones in foxtail millet leaves is significantly positively correlated with antioxidant system indexes, while IAA and GA contents are significantly negatively correlated with antioxidant system indexes. In conclusion, after penoxsulam treatment, foxtail millet may regulate the defense ability of the antioxidant system by regulating the content of endogenous hormones, thereby enhancing the tolerance to herbicide stress. However, this conclusion is only inferred based on correlation analysis and phenotypic observation results, and the causal relationship between endogenous hormones and antioxidant system regulation needs to be further verified by follow-up experiments such as exogenous hormone supplementation or gene editing.

## Figures and Tables

**Table 1:** Herbicide dosage/g a.i. Ha^−1^.

Variety	Treatment			
	P_0_	P_1/2_	P_1_	P_2_
Jingu 21	0	15	30	60

**Table 2:** Nutrient content of 0–20 cm soil layer before sowing in 2024.

Year	Total P (g/kg)	Total K (g/kg)	Total N (g/kg)	Available K (mg/kg)	Available N (mg/kg)	Available P (mg/kg)	Organic Matter (g/kg)	pH
2024	1.058	22.6	1.053	307	70.4	12.4	24.4	8.16

## Data Availability

The original contributions presented in this study are included in the article. Further inquiries can be directed to the corresponding authors.

## Supplementary Materials

The complete raw experimental data can be downloaded at: https://www.mdpi.com/article/10.3390/plants14213254/s1, Table S1. IAA content of foxtail millet. Table S2. GA content of foxtail millet. Table S3. ABA content of foxtail millet. Table S4. H2O2 content of foxtail millet. Table S5. O2− content of foxtail millet. Table S6. GSH content of foxtail millet. Table S7. MP of foxtail millet.
