# Supplementary material for: Regulatory Effects of Different Doses of Penoxsulam on Endogenous Hormones and Antioxidant System in Foxtail Millet
Source: Plants (Basel). 2025 Oct 24;14(21):3254. doi: 10.3390/plants14213254 (PMC12610494; doi:10.3390/plants14213254)
Supplement: Supplementary file 1 [file plants-14-03254-s001.zip › plants-3860405-supplementary.pdf]

Table S1

Treatments	Days after application / d							
	1d	2d	3d	5d	10d	7d	14d	28d
CK-1	42.31	47.04	47.57	55.13	64.29	93.60	119.40	112.92
CK-2	43.07	47.04	47.57	55.13	64.29	95.66	119.40	110.70
CK-3	42.99	45.82	48.18	55.82	65.89	95.66	119.94	110.70
CK-4	43.30	47.11	46.12	54.21	63.45	96.20	118.26	114.06
1/2X-1	42.53	46.35	58.03	67.88	70.47	102.00	123.91	114.67
1/2X-2	42.53	46.35	58.03	67.88	70.47	102.00	123.91	114.67
1/2X-3	42.23	45.89	59.10	68.79	72.69	104.75	124.52	114.06
1/2X-4	42.61	45.36	57.27	67.04	69.02	100.55	125.36	118.34
1X-1	42.38	42.46	44.75	50.40	51.69	92.61	117.11	106.12
1X-2	42.38	42.46	44.75	50.40	51.69	92.61	117.11	106.12
1X-3	40.47	41.16	44.21	48.79	51.16	93.91	118.34	106.73
1X-4	41.47	43.37	45.05	51.47	52.53	91.16	115.82	106.96
2X-1	41.77	41.54	42.76	49.18	50.85	81.31	114.29	101.62
2X-2	41.77	41.54	42.76	49.18	50.85	81.31	114.29	101.62
2X-3	41.31	42.08	43.45	48.72	49.63	78.64	115.36	111.47
2X-4	42.23	42.53	42.99	48.18	51.77	82.00	114.37	100.55

Table S2

Treatments	Days after application / d							
	1d	2d	3d	5d	10d	7d	14d	28d
CK-1	605.54	645.71	687.68	685.00	721.61	698.39	827.86	787.68
CK-2	605.54	645.71	687.68	685.00	721.61	698.39	827.86	787.68
CK-3	609.11	633.21	695.71	695.71	713.57	703.75	837.68	792.14
CK-4	598.39	652.86	682.32	693.04	720.71	690.36	822.50	779.64
1/2X-1	583.21	585.00	712.68	726.07	735.00	743.93	846.61	794.82
1/2X-2	583.21	585.00	712.68	726.07	735.00	743.93	846.61	794.82
1/2X-3	568.93	570.71	705.54	714.46	738.57	748.39	858.21	798.39
1/2X-4	588.57	595.71	718.93	731.43	722.50	735.00	839.46	787.68
1X-1	573.39	583.21	653.75	656.43	701.96	664.46	819.82	781.43
1X-2	573.39	583.21	653.75	656.43	710.89	664.46	819.82	781.43
1X-3	580.54	586.79	643.04	680.54	715.36	652.86	816.25	787.68
1X-4	590.36	580.54	658.21	678.75	720.71	668.93	828.75	771.61
2X-1	565.36	577.86	624.29	669.82	699.29	616.25	817.14	776.96
2X-2	565.36	577.86	624.29	669.82	699.29	616.25	817.14	776.96
2X-3	571.61	574.29	613.57	660.00	705.54	613.57	822.50	762.68
2X-4	579.64	591.25	630.54	651.96	702.86	623.39	807.32	784.11

Table S3

Treatments	Days after application / d							
	1d	2d	3d	5d	10d	7d	14d	28d
CK-1	196.02	219.75	240.59	268.56	320.42	229.92	301.44	347.54
CK-2	196.02	219.75	240.59	268.56	320.42	229.92	301.44	347.54
CK-3	198.39	220.76	237.20	262.80	322.29	223.98	303.81	350.59
CK-4	197.54	217.71	242.63	269.58	322.97	227.54	302.12	342.97
1/2X-1	200.42	225.17	249.07	265.51	323.64	233.98	303.47	349.92
1/2X-2	200.42	225.17	249.07	265.51	323.64	233.98	303.47	349.92
1/2X-3	201.78	229.07	250.76	261.44	329.24	236.36	305.00	347.71
1/2X-4	198.73	223.47	246.02	265.22	320.76	230.42	302.29	344.83
1X-1	210.25	220.76	260.25	281.10	353.47	251.44	323.47	360.76
1X-2	210.25	220.76	260.25	281.10	353.47	251.44	323.47	360.76
1X-3	206.86	219.75	263.31	279.58	355.85	253.47	322.63	356.02
1X-4	213.98	222.46	257.37	282.29	349.92	249.07	320.08	358.39
2X-1	213.64	241.95	277.37	288.22	358.22	255.68	331.95	370.42
2X-2	213.64	241.95	277.37	288.22	358.22	255.68	331.95	370.42
2X-3	211.10	246.36	281.61	290.25	356.86	253.98	329.24	364.49
2X-4	207.37	239.07	275.00	287.37	361.61	259.41	328.05	363.47

Table S4

Treatments	Days after application / d							
	1d	2d	3d	5d	10d	7d	14d	28d
CK-1	0.64	0.66	0.79	0.88	1.37	1.34	1.84	1.91
CK-2	0.64	0.66	0.79	0.88	1.37	1.34	1.84	1.91
CK-3	0.69	0.71	0.81	0.93	1.38	1.33	1.85	1.87
CK-4	0.63	0.69	0.77	0.88	1.36	1.37	1.81	1.93
1/2X-1	0.69	0.77	0.82	0.86	1.56	1.72	1.85	1.88
1/2X-2	0.69	0.77	0.82	0.86	1.56	1.72	1.85	1.88
1/2X-3	0.74	0.74	0.85	0.92	1.61	1.80	1.87	1.86
1/2X-4	0.68	0.72	0.80	0.86	1.54	1.69	1.81	1.90
1X-1	0.72	0.87	0.93	0.97	1.61	1.94	2.20	2.26
1X-2	0.72	0.87	0.93	0.97	1.61	1.94	2.20	2.26
1X-3	0.69	0.89	0.98	0.95	1.60	1.87	2.19	2.19
1X-4	0.69	0.84	0.95	0.98	1.65	1.96	2.14	2.24
2X-1	0.74	0.91	1.03	1.12	1.73	2.00	2.26	2.27
2X-2	0.74	0.91	1.03	1.12	1.73	2.00	2.26	2.27
2X-3	0.68	0.87	1.07	1.09	1.75	2.05	2.23	2.31
2X-4	0.75	0.86	1.01	1.15	1.70	1.99	2.28	2.26

Table S5

Treatments	Days after application / d							
	1d	2d	3d	5d	10d	7d	14d	28d
CK-1	0.09	0.10	0.10	0.12	0.16	0.10	0.16	0.16
CK-2	0.09	0.10	0.10	0.12	0.16	0.10	0.16	0.13
CK-3	0.11	0.09	0.09	0.15	0.14	0.09	0.14	0.15
CK-4	0.11	0.12	0.06	0.12	0.17	0.09	0.17	0.15
1/2X-1	0.11	0.11	0.12	0.13	0.16	0.11	0.16	0.15
1/2X-2	0.09	0.11	0.12	0.13	0.16	0.11	0.17	0.17
1/2X-3	0.12	0.11	0.13	0.15	0.18	0.15	0.16	0.13
1/2X-4	0.11	0.13	0.13	0.11	0.19	0.13	0.17	0.16
1X-1	0.12	0.14	0.16	0.16	0.20	0.16	0.21	0.19
1X-2	0.12	0.16	0.16	0.16	0.20	0.16	0.21	0.19
1X-3	0.13	0.15	0.16	0.20	0.21	0.17	0.22	0.20
1X-4	0.10	0.14	0.18	0.21	0.18	0.16	0.22	0.18
2X-1	0.11	0.14	0.18	0.21	0.20	0.19	0.23	0.19
2X-2	0.11	0.16	0.18	0.21	0.23	0.19	0.23	0.19
2X-3	0.13	0.16	0.18	0.18	0.24	0.20	0.24	0.20
2X-4	0.14	0.14	0.16	0.20	0.21	0.17	0.25	0.19

Table S6

Treatments	Days after application / d							
	1d	2d	3d	5d	10d	7d	14d	28d
CK-1	199.16	196.36	199.96	226.76	182.36	195.96	217.56	206.36
CK-2	160.76	196.36	199.96	192.36	185.16	182.76	216.36	191.56
CK-3	196.36	202.76	198.76	193.16	221.56	202.36	231.56	199.16
CK-4	198.36	185.16	201.16	223.56	219.56	191.56	241.56	195.16
1/2X-1	188.76	207.96	241.96	226.76	250.76	211.96	243.96	223.16
1/2X-2	191.96	207.96	241.96	268.36	254.36	206.76	242.76	218.36
1/2X-3	188.76	206.76	243.16	265.56	251.96	199.56	262.76	207.16
1/2X-4	187.96	209.16	240.76	266.36	253.96	211.16	239.16	222.36
1X-1	205.96	222.76	267.96	286.76	251.96	233.16	250.76	232.76
1X-2	203.96	182.76	267.96	285.16	255.96	232.36	248.36	232.36
1X-3	204.36	221.56	282.76	250.36	249.56	241.16	262.76	240.76
1X-4	205.96	203.96	269.16	289.96	251.56	230.36	242.36	220.36
2X-1	222.36	239.16	281.96	299.56	295.96	263.96	273.16	239.96
2X-2	222.36	227.16	282.36	301.16	296.76	262.76	272.36	249.96
2X-3	224.36	233.96	279.56	338.36	294.36	272.36	279.56	245.56
2X-4	241.16	243.16	280.76	299.96	297.16	281.96	281.16	246.36

Table S7

Treatments	Days after application / d							
	1d	2d	3d	5d	10d	7d	14d	28d
CK-1	69.46%	67.93%	72.15%	67.44%	69.16%	81.68%	80.64%	53.49%
CK-2	50.92%	71.60%	73.29%	77.92%	80.46%	77.22%	77.39%	68.91%
CK-3	73.10%	61.36%	71.69%	75.47%	83.47%	76.72%	74.16%	50.53%
CK-4	70.31%	65.08%	65.84%	77.55%	71.24%	82.91%	76.34%	50.57%
1/2X-1	82.03%	81.97%	77.89%	92.93%	85.34%	83.71%	81.00%	63.71%
1/2X-2	65.09%	68.35%	79.66%	84.42%	83.69%	78.34%	81.34%	35.48%
1/2X-3	57.88%	67.75%	79.98%	83.57%	83.39%	80.48%	71.74%	76.50%
1/2X-4	65.84%	74.57%	83.45%	83.07%	80.17%	82.77%	81.37%	69.64%
1X-1	74.91%	86.69%	84.01%	86.84%	82.08%	83.75%	85.32%	85.34%
1X-2	67.29%	76.35%	87.90%	91.40%	85.81%	84.62%	83.78%	79.43%
1X-3	77.14%	75.86%	86.45%	91.47%	81.29%	84.67%	79.12%	80.04%
1X-4	72.31%	85.82%	81.43%	78.33%	79.74%	85.26%	84.84%	82.45%
2X-1	71.38%	89.49%	89.40%	93.19%	87.45%	87.39%	84.19%	84.72%
2X-2	74.76%	88.09%	87.54%	95.09%	88.17%	85.36%	84.85%	83.01%
2X-3	78.11%	82.56%	92.84%	93.95%	87.49%	84.60%	82.95%	79.70%
2X-4	82.86%	82.97%	89.42%	94.80%	86.01%	86.27%	98.00%	79.34%
